# Supplementary material for: Methylation profiles at birth linked to early childhood obesity
Source: J Dev Orig Health Dis. Author manuscript; Available in PMC 2024 Oct 25. (PMC11268442; doi:10.1017/S2040174424000060)
Supplement: 1 [file NIHMS1967709-supplement-1.docx]

**Supplemental Information:**

**Methylation profiles at birth linked to early childhood obesity**

Delphine Lariviere^1*^, Sarah J.C. Craig^2,3*^, Ian M. Paul^3,4^, Emily E. Hohman^5^, Jennifer S. Savage^5,6^, Robert O. Wright^7^, Francesca Chiaromonte^3,8,9#^, Kateryna D. Makova^2,3#^, and Matthew L. Reimherr^3,8#^

##

1. Department of Biochemistry and Molecular Biology, Penn State University, University Park, PA
2. Department of Biology, Penn State University, University Park, PA
3. Center for Medical Genomics, Penn State University, University Park, PA
4. Department of Pediatrics, Penn State College of Medicine, Hershey, PA
5. Center for Childhood Obesity Research, Penn State University, University Park, PA
6. Nutrition Department, Penn State University, University Park, PA
7. Icahn School of Medicine, Mount Sinai, New York, NY
8. Department of Statistics, Penn State University, University Park, PA
9. EMbeDS, Sant’Anna School of Advanced Studies, Piazza Martiri della Libertà, Pisa, Italy

* These authors have contributed equally

# Corresponding authors

**Table of Contents:**

[**Supplementary Figure 1. Phenotype distribution 4**](#_q1vmgeue6vok)

[**Supplementary Figure 2. BMI standardization LASSO result 5**](#_cohtcodhqieb)

[**Supplementary Figure 3. Preprocessing workflow 6**](#_n1f0apcp4b9c)

[**Supplementary Figure 4. Methylation analysis workflow 7**](#_1xyq4pqpuing)

[**Supplementary Figure 5. Methylation LASSO results 8**](#_z2r6yb8vkk8y)

[**Supplementary Table 1. Number of CpGs included at each step of the analysis 9**](#_t80v25c6iqz6)

[**Supplementary Table 2. Parameters used for LASSO Analyses. 10**](#_4goeejutbyjf)

[**Supplementary Table 3. Genes identified in differential methylation analysis of conditional weight gain (CWG) in cord blood ordered from highest to lowest Ordinary Least Squares(OLS) coefficients. 11**](#_o0ysxkhdd5zx)

[**Supplementary Table 4. Genes identified in differential methylation analysis of Body Mass Index (BMI) in cord blood ordered from highest to lowest OLS coefficients. 12**](#_8ka4cfdhtuhq)

[**Supplementary Table 5. Genes identified in differential methylation analysis of weight/length ratio in cord blood ordered from highest to lowest OLS coefficient. 13**](#_ciyn59mbt7xf)

[**Supplementary Table 6. Genes identified in differential methylation analysis of conditional weight gain (CWG) in placenta ordered from highest to lowest OLS coefficient. 15**](#_qi31nuhsw2ez)

[**Supplementary Table 7. Regression coefficients and p-values from PROGRESS validation dataset using Conditional Weight Gain. 16**](#_5an432yx46ut)

[**Supplementary Table 8. Regression coefficients and p-values from PROGRESS validation dataset using Body Mass Index. 17**](#_8a3yax9jrlgc)

[**Supplementary Table 9. Regression coefficients and p-values from PROGRESS validation dataset using Weight-for-Length. 18**](#_ejwp2ivpjs5)

[**Supplementary Table 10. Methylation Risk Score associations with phenotypes at later time points in the SIBSIGHT cohort. 19**](#_79pz6n934vlh)

[**Supplementary Table 11. Methylation Risk Score Associations with phenotypes in the PROGRESS cohort. 20**](#_q5js2txagu6h)

[**Supplementary Note 1. Stratified analysis based on sex 21**](#_k5cy864bww5w)

[Females - Cord Blood 22](#_471hxpb9jsyz)

[Genes associated with CWG 22](#_jvyy9pazpt0u)

[Genes associated with BMI 22](#_hjkpqm69u61m)

[Genes associated with the weight-for-length ratio 23](#_lllh084p3gmv)

[Males - Cord Blood 23](#_v7hto4xgy82w)

[Genes associated with CWG 23](#_78usckw491c3)

[Genes associated with BMI 23](#_mj1zyaa7f0l2)

[Genes associated with the weight-for-length ratio 23](#_ieotcn497llb)

[Females - Placenta 24](#_mk6ntdp05dar)

[Genes associated with CWG 24](#_3ihx03uio9q4)

[Genes associated with BMI 25](#_769luerusvo)

[Genes associated with the weight-for-length ratio 25](#_76wgfidob0sw)

[Males - Placenta 25](#_msq8zoootfz)

[Genes associated with CWG 25](#_z8zmdee3ohk6)

[Genes associated with BMI 25](#_umeejfbln443)

[Genes associated with the weight-for-length ratio 25](#_j90oyhi87bj4)

[**Supplementary Note 2. Location of associated CpG sites in associated genes 27**](#_xsy7fgnbvns2)

[CpGs Associated with CWG in Cord Blood 29](#_6eyl24l3u1ti)

[CpGs Associated with BMI in Cord Blood 29](#_ts28eewb5ntp)

[CpGs Associated with CWG in Placenta 30](#_ziauuf3ixjed)

[**Supplementary Note 3. Evaluating the methylation effects of genes previously identified to contain genetic variants important for childhood obesity 32**](#_nh900az68vrb)

# **Supplementary Figure 1. Phenotype distribution**

**Distribution of (A)** CWG *z*-score from 0 to 6 months, (**B)** weight-for-length at six months, (**C)** BMI at the age of six months.

**
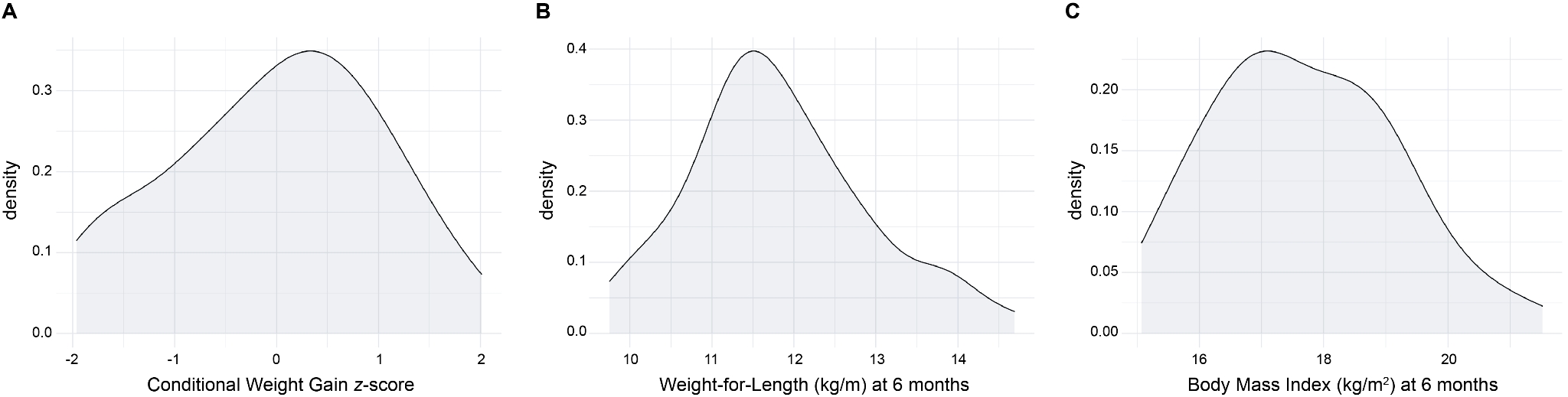
**

# **Supplementary Figure 2. BMI standardization LASSO result**

LASSO analysis to measure the association between covariates and BMI after standardization per sex. The absence of a stable minimum indicates no significant correlation between any other covariates and the phenotype.
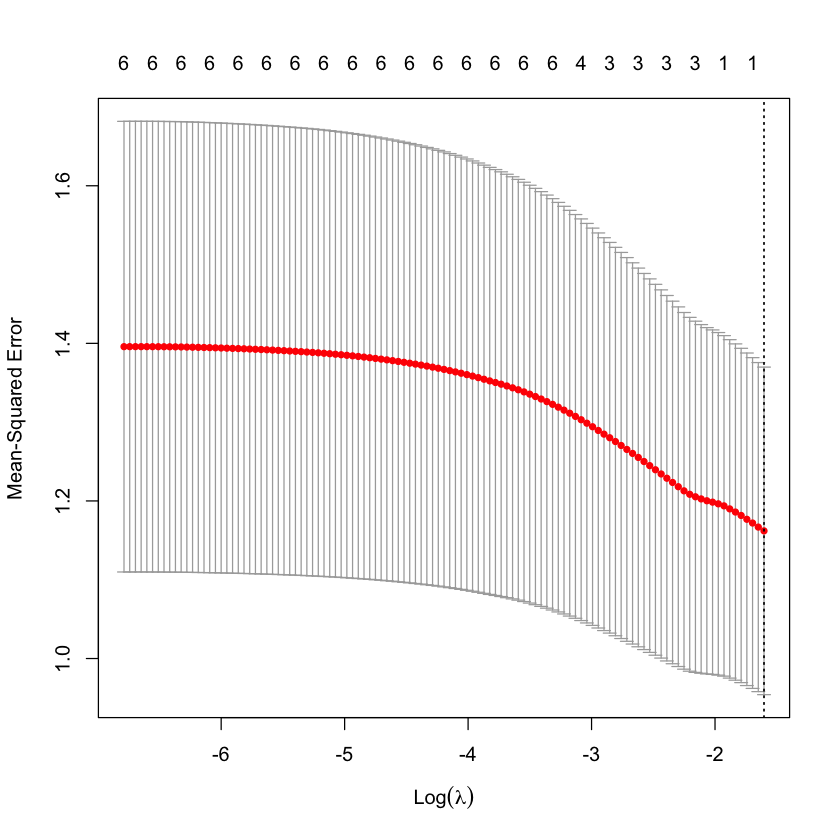


# **Supplementary Figure 3. Preprocessing workflow**

Workflow depicting the preprocessing of chip signal data. **(A)** Signal treatment and quality control. The raw data contain light intensity signals for each cell. The first step is to convert the light signal to methylation state of the corresponding CpG site, then to remove data that are of poor quality or that could artificially impact the association study such as sex chromosomes or probes with known SNPs. **(B)** Data normalization. Two types of normalizations are necessary. The first one normalizes the signal within each chip type. The second one normalizes between the two types of chips, allowing them to be used together in the analysis.


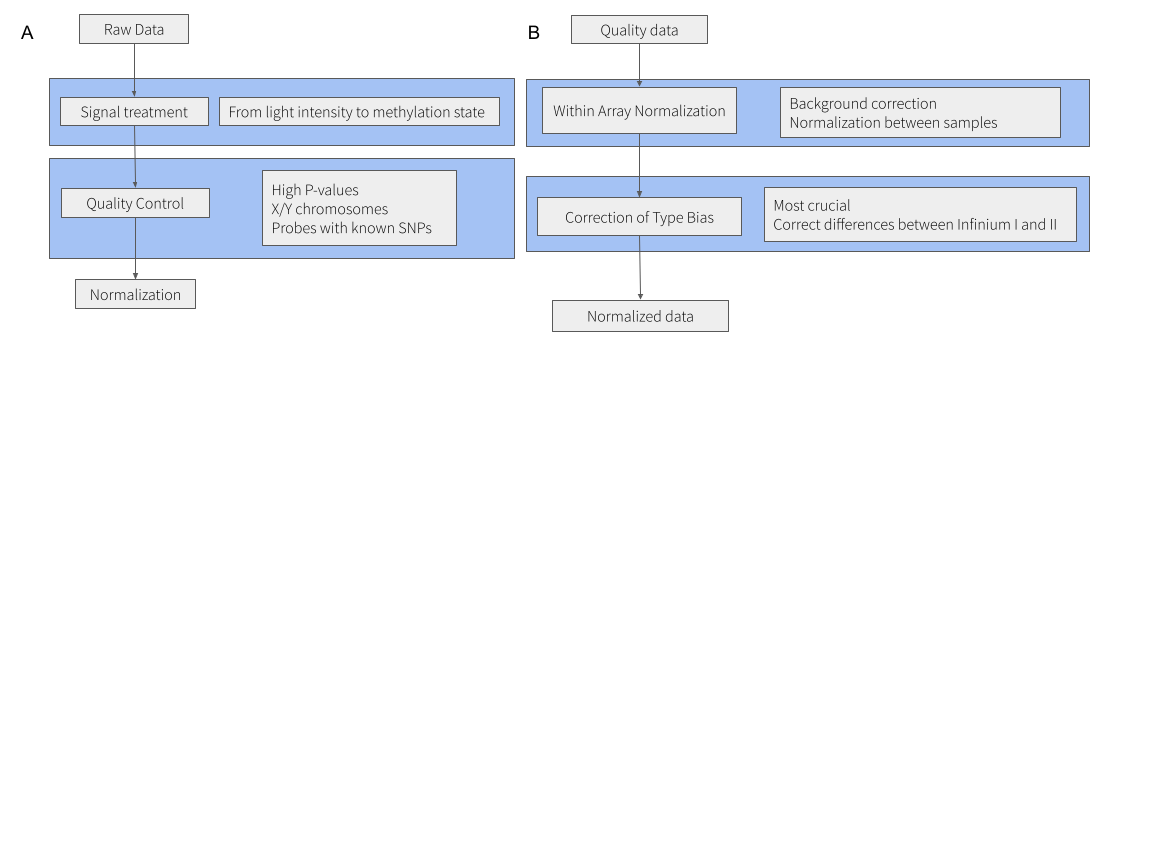


# **Supplementary Figure 4. Methylation analysis workflow**

Workflow depicting the association study to identify gene methylation profiles linked to weight outcome in children. The left side of the figure describes attempts to identify individual differentially methylated CpGs, the results of which were inconclusive. The right side of the figure describes the workflow we followed by grouping the CpGs by gene.


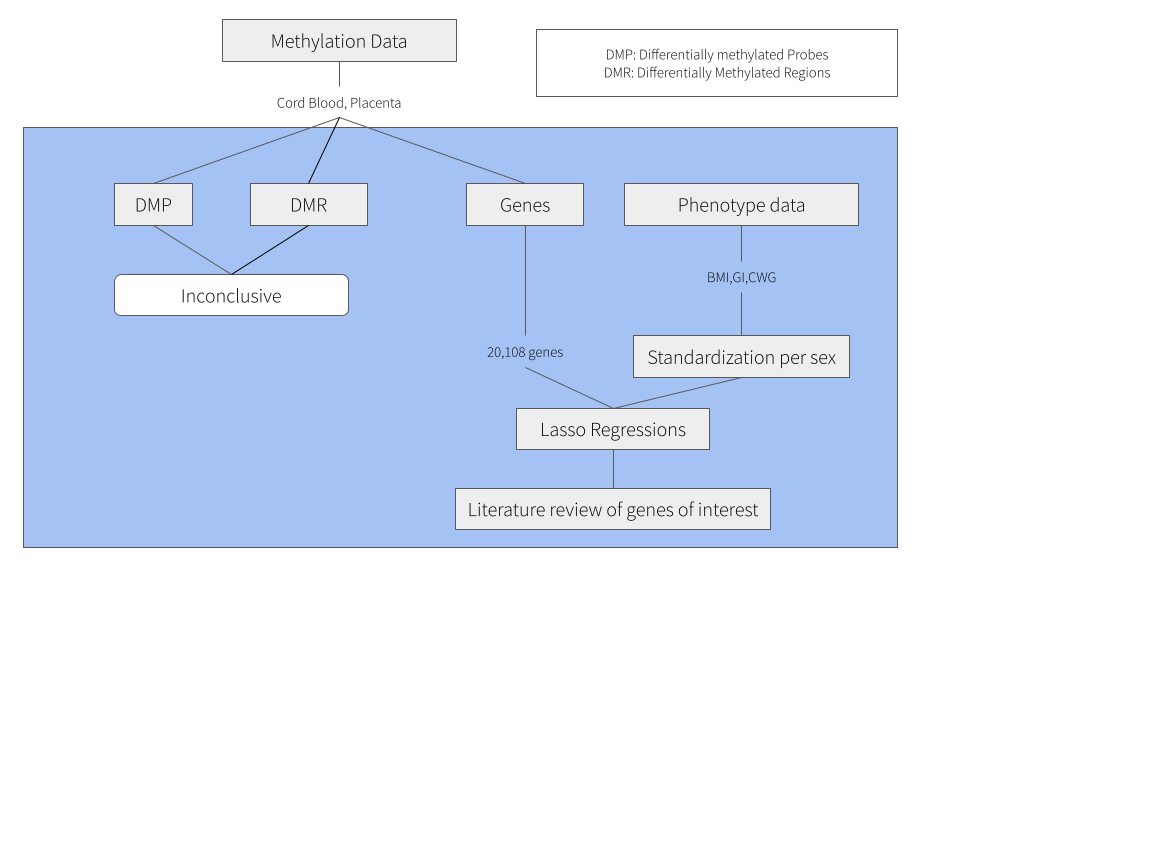


#

# **Supplementary Figure 5.** **Methylation LASSO results**

LASSO analysis to identify methylation profiles of genes linked to infant weight gain. (**A)** Methylated genes in cord blood associated with CWG. The LASSO analysis identified seven genes associated with CWG with a Mean-Squared error around 1. (**B)** Methylated genes in the placenta associated with CWG. The LASSO analysis identified ten genes associated with CWG with a Mean-Squared error above 1. (**C)** Methylated genes in cord blood associated with the BMI. The LASSO analysis identified four genes associated with CWG with a Mean-Squared error above 2.2. (**D)** Methylated genes in placenta associated with the BMI. The LASSO analysis did not identify methylation to be associated with the BMI. (**E)** Methylated genes in the blood cord associated with the weight-for-length ratio. The LASSO analysis identified 27 gene methylations linked to the weight-for-length ratio, but the Mean-Squared error curve was almost flat and around 1.3. (**F)** Methylated genes in the placenta associated with the weight-for-length ratio. The LASSO analysis did not identify methylation to be associated with the weight-for-length ratio.


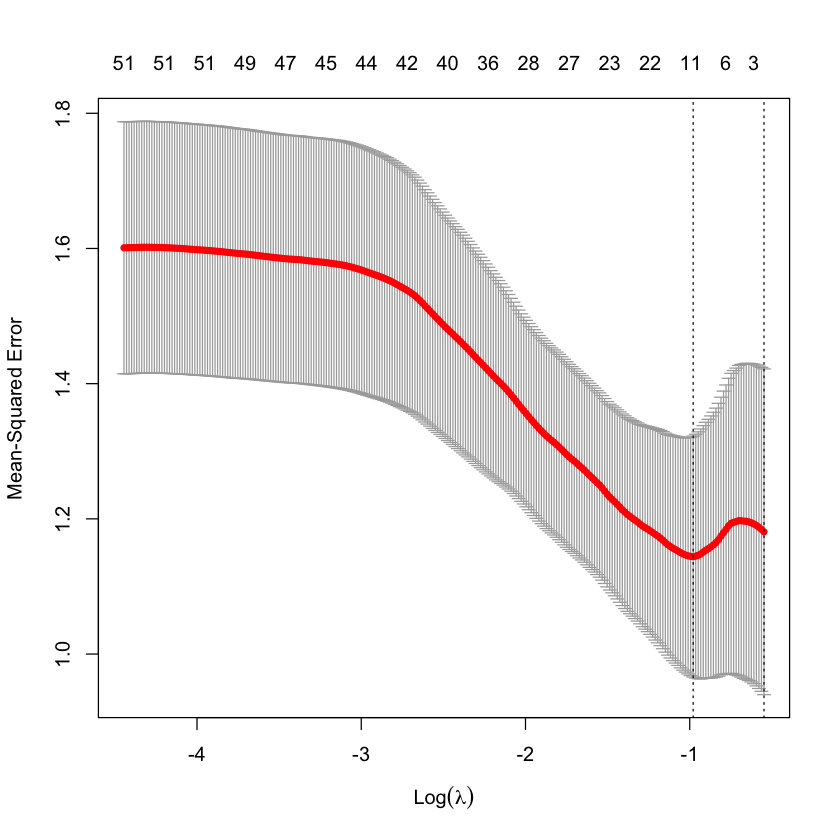

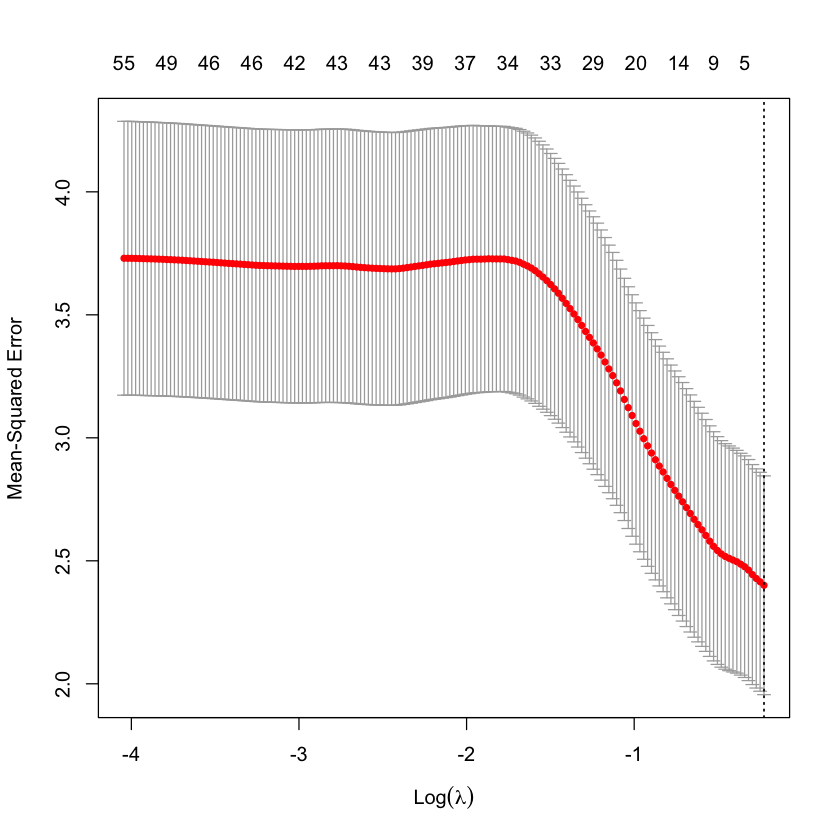

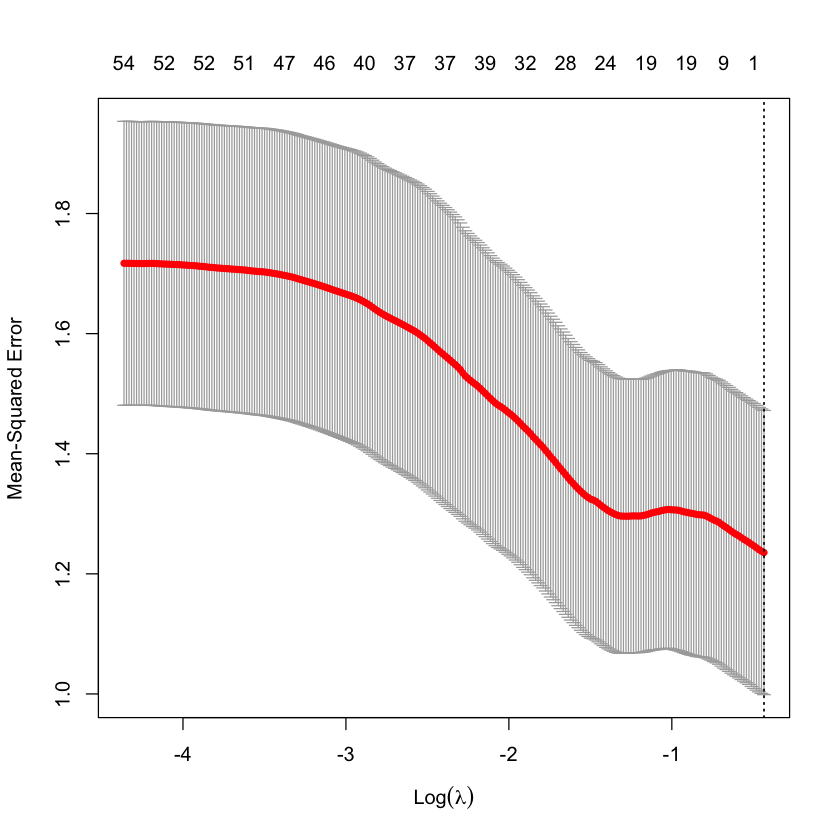


CWG

Placenta

Cord Blood

BMI

GI

A

B

C

D

E

F


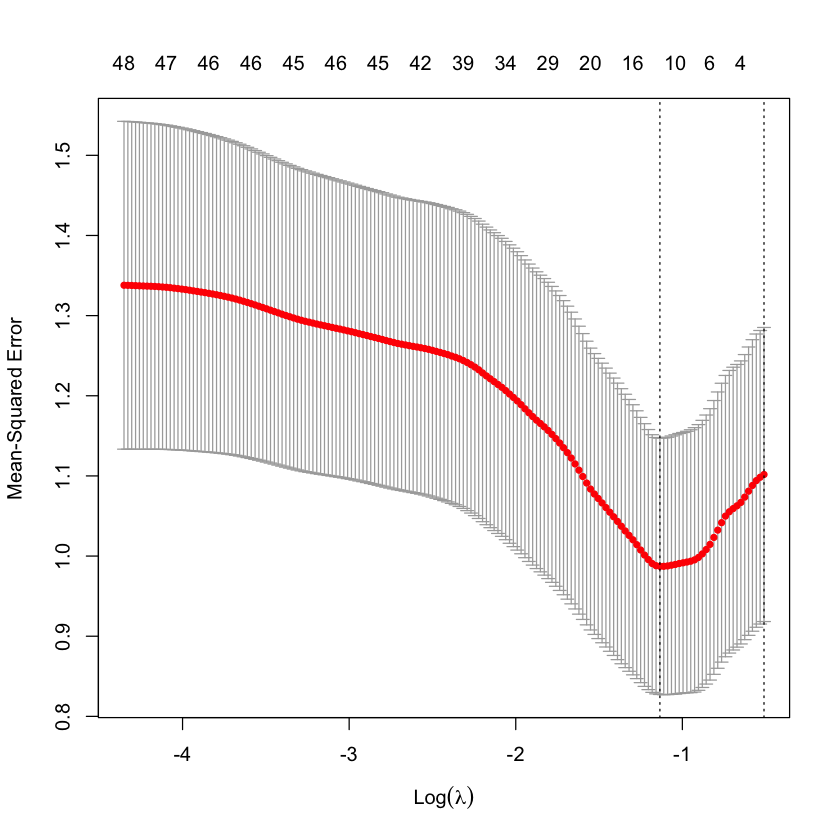

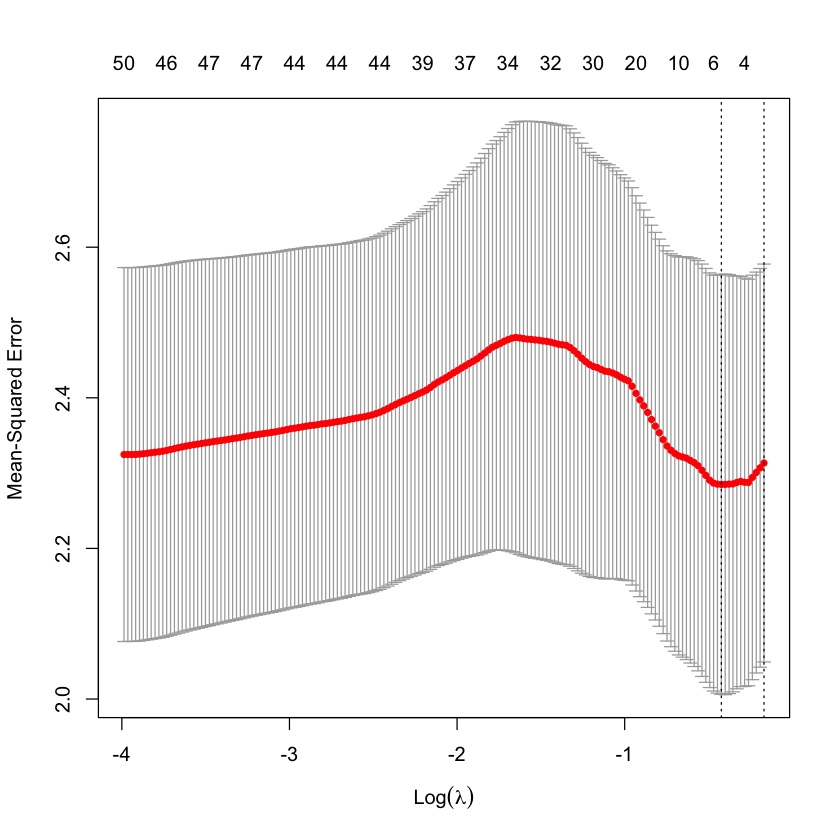

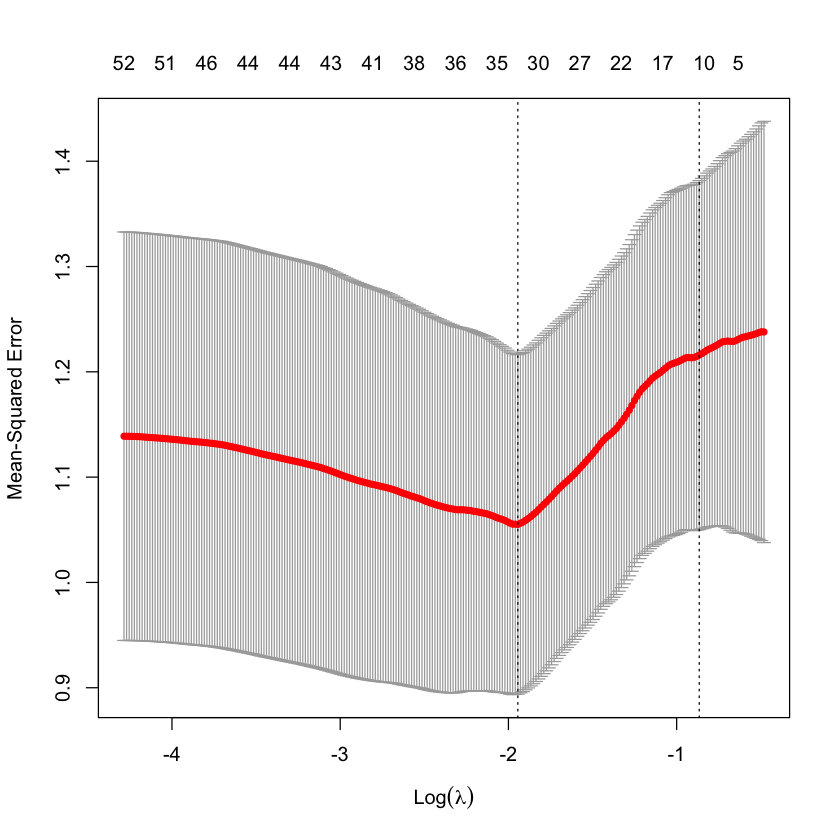


# **Supplementary Table 1.** Number of CpGs included at each step of the analysis

| Tissue | Step | Number of CpGs remaining |
| --- | --- | --- |
| Placenta | Initial | 1,027,699 |
|  | Post quality control | 575,132 |
|  | Removing outliers and insufficient samples | 452,567 |
|  | Removal of CpGs associated with common SNPs | 436,251 |
|  | CpGs in genes | 293,090 |
| Cord Blood | Initial | 1,027,699 |
|  | Post quality control | 575,132 |
|  | Removal outliers and insufficient samples | 452,567 |
|  | Removal of CpGs associated with common SNPs | 436,251 |
|  | CpGs in genes | 293,090 |

# **Supplementary Table 2**. Parameters used for LASSO Analyses.

| Analysis | Nfold^1^ | alpha^2^ | nlambda^3^ |
| --- | --- | --- | --- |
| Covariates | 10 | 1 | 200 |
| CWG Placenta | 10 | 1 | 300 |
| CWG Cord Blood | 10 | 1 | 200 |
| BMI Placenta | 10 | 1 | 200 |
| BMI Cord Blood | 10 | 1 | 200 |
| weight-for-length ratio Placenta | 10 | 1 | 300 |
| weight-for-length ratio Cord Blood | 10 | 1 | 300 |

^1^ The number of folds to be used in the cross-validation

^2^ Elastic net mixing parameter. alpha=1 is lasso regression (default) and alpha=0 is ridge regression

^3^ The number of 𝜆 values to be tested

###

# **Supplementary Table 3.** Genes identified in differential methylation analysis of conditional weight gain (CWG) in cord blood ordered from highest to lowest Ordinary Least Squares(OLS) coefficients.

| **Name** | **Chr** | **start** | **end** | **Function** | **Marginal regression Coefficient** | **Marginal p-value** | **Correlation Coefficient** | **OLS coefficient** | **OLS p-value** |
| --- | --- | --- | --- | --- | --- | --- | --- | --- | --- |
| *LAMP3* | chr3 | 183122215 | 183162761 | lysosomal associated membrane protein 3 | 118.29 | 8.39E-05 | 0.54 | 28.84 | 8.61E-02 |
| *EARS2* | chr16 | 23520754 | 23557375 | glutamyl-tRNA synthetase 2%2C mitochondrial | 94.89 | 2.44E-04 | 0.51 | 23.63 | 1.22E-01 |
| *CCDC28A-AS1* | chr6 | 138725215 | 138773703 | CCDC28A antisense RNA 1 | 76.86 | 1.26E-05 | 0.58 | 23.49 | 3.29E-02 |
| *PLIN4* | chr19 | 4502192 | 4520285 | perilipin 4 | 45.45 | 1.33E-05 | 0.58 | 13.25 | 5.36E-02 |
| *UBE2F* | chr2 | 237966945 | 238042782 | ubiquitin conjugating enzyme E2 F (putative) | 93.30 | 3.07E-05 | 0.56 | 11.88 | 3.49E-01 |
| *STAP1* | chr4 | 67558727 | 67607337 | signal transducing adaptor family member 1 | 27.14 | 1.18E-03 | 0.45 | 9.33 | 4.82E-02 |
| *YARS2* | chr12 | 32725247 | 32756458 | tyrosyl-tRNA synthetase 2 | -12.91 | 9.17E-04 | -0.46 | -3.27 | 1.11E-01 |
| *PPP1R16B* | chr2 | 38805694 | 38923024 | protein phosphatase 1 regulatory subunit 16B | -71.25 | 1.71E-05 | -0.58 | -12.19 | 2.50E-01 |
| *TLK1* | chr2 | 170990823 | 171231293 | tousled like kinase 1 | -29.16 | 2.72E-03 | -0.42 | -15.59 | 2.52E-03 |
| *ANKS4B* | chr16 | 21233699 | 21253850 | ankyrin repeat and sterile alpha motif domain containing 4B | -27.62 | 1.55E-04 | -0.52 | -16.81 | 4.00E-05 |
| *LINC00486* | chr2 | 32825433 | 32946136 | long intergenic non-protein coding RNA 486 | -90.42 | 1.06E-03 | -0.46 | -24.12 | 9.89E-02 |

# **Supplementary Table 4.** Genes identified in differential methylation analysis of Body Mass Index (BMI) in cord blood ordered from highest to lowest OLS coefficients.

| **Name** | **Chr** | **start** | **end** | **Function** | **Marginal regression Coefficient** | **Marginal p-value** | **Correlation Coefficient** | **OLS coefficient** | **OLS p-value** |
| --- | --- | --- | --- | --- | --- | --- | --- | --- | --- |
| *UBE2F* | chr2 | 237966945 | 238042782 | ubiquitin conjugating enzyme E2 F (putative) | 133.19 | 4.02E-05 | 0.56 | 45.07 | 1.14E-01 |
| *PLIN4* | chr19 | 4502192 | 4520285 | perilipin 4 | 64.05 | 2.41E-05 | 0.57 | 37.17 | 3.79E-03 |
| *HRH2* | chr5 | 175657762 | 175710756 | histamine receptor H2 | 54.76 | 6.58E-05 | 0.54 | 25.23 | 2.70E-02 |
| *PPP1R16B* | chr2 | 38805694 | 38923024 | protein phosphatase 1 regulatory subunit 16B | -98.27 | 4.88E-05 | -0.55 | -56.98 | 5.50E-03 |

#

# **Supplementary Table 5.** Genes identified in differential methylation analysis of weight/length ratio in cord blood ordered from highest to lowest OLS coefficient.

| **Name** | **Chr** | **start** | **end** | **Function** | **Marginal regression Coefficient** | **Marginal p-value** | **Correlation Coefficient** | **OLS coefficient** | **OLS p-value** |
| --- | --- | --- | --- | --- | --- | --- | --- | --- | --- |
| *SMIM20* | chr4 | 25914192 | 25929879 | small integral membrane protein 20 | 96.04 | 2.76E-03 | 0.42 | 21.77 | 3.02E-02 |
| *ZFP90* | chr16 | 68530249 | 68576072 | ZFP90 zinc finger protein | 146.46 | 1.14E-04 | 0.53 | 19.83 | 8.83E-02 |
| *LOC105376031* | chr9 | 37027763 | 37031333 |  | 65.46 | 5.27E-04 | 0.48 | 15.53 | 4.38E-03 |
| *ERP27* | chr12 | 14914027 | 14938537 | endoplasmic reticulum protein 27 | 56.14 | 1.36E-03 | 0.45 | 10.64 | 3.03E-02 |
| *MFSD3* | chr8 | 144508081 | 144511228 | major facilitator superfamily domain containing 3 | 108.96 | 8.27E-04 | 0.47 | 10.64 | 2.67E-01 |
| *HMGCS1* | chr5 | 43287470 | 43313477 | 3-hydroxy-3-methylglutaryl-CoA synthase 1 | 55.18 | 2.40E-05 | 0.57 | 9.31 | 3.49E-02 |
| *DNASE1* | chr16 | 3611737 | 3665472 | deoxyribonuclease 1 | 65.46 | 7.46E-03 | 0.38 | 9.26 | 1.98E-01 |
| *LOC105369771* | chr12 | 52105711 | 52119039 |  | 47.54 | 3.34E-05 | 0.56 | 8.92 | 7.50E-03 |
| *BMF* | chr15 | 40087890 | 40108879 | Bcl2 modifying factor | 36.11 | 8.76E-05 | 0.54 | 7.06 | 2.33E-02 |
| *KCTD20* | chr6 | 36442767 | 36491143 | potassium channel tetramerization domain containing 20 | 15.05 | 1.99E-03 | 0.44 | 6.92 | 2.76E-05 |
| *PLIN4* | chr19 | 4502192 | 4520285 | perilipin 4 | 39.42 | 5.76E-04 | 0.48 | 2.86 | 5.25E-01 |
| *ABHD16A* | chr6 | 31686955 | 31703324 | abhydrolase domain containing 16A | 98.11 | 1.78E-04 | 0.52 | 2.47 | 7.48E-01 |
| *CCNG2* | chr4 | 77157207 | 77170060 | cyclin G2 | 14.56 | 3.20E-03 | 0.42 | 2.29 | 4.23E-02 |
| *CCDC28A-AS1* | chr6 | 138725215 | 138773703 | CCDC28A antisense RNA 1 | 65.40 | 7.38E-04 | 0.47 | 2.17 | 7.20E-01 |
| *GZF1* | chr2 | 23361585 | 23375399 | GDNF inducible zinc finger protein 1 | -11.06 | 3.01E-04 | -0.50 | -0.95 | 2.82E-01 |
| *YARS2* | chr12 | 32725247 | 32756458 | tyrosyl-tRNA synthetase 2 | -14.27 | 4.90E-04 | -0.48 | -1.54 | 2.24E-01 |
| *LOC107984670* | chr14 | 104741144 | 104741840 |  | -24.62 | 3.96E-03 | -0.41 | -1.54 | 4.87E-01 |
| *LMTK2* | chr7 | 98106862 | 98209638 | lemur tyrosine kinase 2 | -24.29 | 1.08E-03 | -0.46 | -2.24 | 2.21E-01 |
| *FAM168B* | chr2 | 131047876 | 131093468 | family with sequence similarity 168 member B | -10.75 | 2.79E-03 | -0.42 | -2.30 | 2.73E-02 |
| *MMP27* | chr11 | 102690943 | 102705785 | matrix metallopeptidase 27 | -23.01 | 9.51E-04 | -0.46 | -3.71 | 1.29E-01 |
| *SLC6A2* | chr16 | 55655928 | 55706192 | solute carrier family 6 member 2 | -70.99 | 3.57E-03 | -0.41 | -4.95 | 4.01E-01 |
| *NCAPH* | chr2 | 96335766 | 96377091 | non-SMC condensin I complex subunit H | -95.21 | 2.28E-04 | -0.51 | -5.87 | 4.37E-01 |
| *RSF1* | chr11 | 77660009 | 77872232 | remodeling and spacing factor 1 | -117.51 | 3.05E-03 | -0.42 | -6.17 | 5.53E-01 |
| *RBM28* | chr7 | 128297685 | 128343915 | RNA binding motif protein 28 | -40.54 | 1.67E-04 | -0.52 | -10.03 | 1.04E-02 |
| *UBE2F* | chr2 | 237966945 | 238042782 | ubiquitin conjugating enzyme E2 F (putative) | 97.05 | 4.41E-05 | 0.55 | -11.81 | 1.14E-01 |
| *ABRAXAS1* | chr4 | 83459517 | 83485137 | abraxas 1%2C BRCA1 A complex subunit | -66.39 | 4.51E-03 | -0.40 | -12.80 | 5.43E-02 |
| *RASSF1* | chr3 | 50329786 | 50340936 | Ras association domain family member 1 | -143.51 | 4.60E-04 | -0.49 | -14.91 | 1.89E-01 |
| *PPP1R16B* | chr2 | 38805694 | 38923024 | protein phosphatase 1 regulatory subunit 16B | -70.34 | 7.68E-05 | -0.54 | -18.48 | 6.00E-04 |
| *LOC100129931* | chr4 | 7030554 | 7046231 | uncharacterized LOC100129931 | -70.10 | 6.01E-03 | -0.39 | -20.60 | 1.98E-02 |
| *LINC00486* | chr2 | 32825433 | 32946136 | long intergenic non-protein coding RNA 486 | -94.81 | 1.18E-03 | -0.45 | -20.83 | 2.10E-02 |
| *OBI1-AS1* | chr13 | 78054855 | 78617325 | OBI1 antisense RNA 1 | -164.46 | 2.67E-03 | -0.42 | -23.95 | 8.88E-02 |

# **Supplementary Table 6.** Genes identified in differential methylation analysis of conditional weight gain (CWG) in placenta ordered from highest to lowest OLS coefficient.

| **Name** | **Chr** | **start** | **end** | **Function** | **Correlation Coefficient** | **Marginal p-value** | **Marginal regression Coefficient** | **OLS coefficient** | **OLS p-value** |
| --- | --- | --- | --- | --- | --- | --- | --- | --- | --- |
| *CUL4A* | chr13 | 113208193 | 113267108 | cullin 4A | 0.41 | 3.67E+01 | 0.00 | 15.80 | 6.17E-02 |
| *OR4D1* | chr17 | 58155154 | 58156086 | olfactory receptor family 4 subfamily D member 1 | 0.50 | 1.22E+01 | 0.00 | 3.60 | 1.11E-01 |
| *GDAP1L1* | chr2 | 44247099 | 44280937 | ganglioside induced differentiation associated protein 1 like 1 | 0.42 | 8.96E+00 | 0.00 | 2.90 | 1.52E-01 |
| *LOC107984087* | chr3 | 111783563 | 111798595 |  | 0.50 | 9.29E+00 | 0.00 | 1.58 | 4.18E-01 |
| *TRIM63* | chr1 | 26051304 | 26067634 | tripartite motif containing 63 | -0.51 | -2.07E+01 | 0.00 | 1.45 | 7.40E-01 |
| *LINC01393* | chr7 | 115078958 | 115126314 | long intergenic non-protein coding RNA 1393 | 0.51 | 1.38E+01 | 0.00 | 0.46 | 8.82E-01 |
| *ADGRB2* | chr1 | 31727105 | 31764340 | adhesion G protein-coupled receptor B2 | 0.55 | 1.29E+01 | 0.00 | 0.10 | 9.69E-01 |
| *TAS2R38* | chr7 | 141972631 | 141973773 | taste 2 receptor member 38 | -0.56 | -6.55E+00 | 0.00 | -1.15 | 3.13E-01 |
| *LOC107985032* | chr17 | 35537124 | 35552311 |  | -0.54 | -7.28E+00 | 0.00 | -2.88 | 4.46E-02 |
| *TM4SF19-TCTEX1D2* | chr3 | 196316085 | 196338420 | TM4SF19-TCTEX1D2 readthrough %28NMD candidate%29 | -0.46 | -9.87E+00 | 0.00 | -3.10 | 1.49E-01 |
| *BTBD18* | chr11 | 57743514 | 57753176 | BTB domain containing 18 | -0.56 | -9.11E+00 | 0.00 | -3.81 | 2.91E-02 |
| *LOC105370500* | chr14 | 52791777 | 52930234 |  | -0.52 | -1.68E+01 | 0.00 | -3.99 | 2.06E-01 |
| *PHC1* | chr12 | 8914509 | 8941467 | polyhomeotic homolog 1 | -0.48 | -1.78E+01 | 0.00 | -4.99 | 1.77E-01 |
| *ACTN1* | chr14 | 68874123 | 68979366 | actinin alpha 1 | -0.52 | -2.74E+01 | 0.00 | -8.17 | 1.34E-01 |

# **Supplementary Table 7.** Regression coefficients and p-values from PROGRESS validation dataset using Conditional Weight Gain.

We used the genes selected in SIBSIGHT as predictors of CWG and performed a linear regression to evaluate their association with PROGRESS CWG scores.

| Name | coefcor^1^ | coeflm^2^ | pvalue^3^ | jointcoeff^4^ | jointpvalue^5^ |
| --- | --- | --- | --- | --- | --- |
| *CCDC28A-AS1* | -0.18786 | -9.2411 | **0.0019341** | -6.1142 | 0.35063 |
| *EARS2* | -0.08657 | -5.7946 | 0.15603 | 3.4249 | 0.53584 |
| *LAMP3* | -0.15740 | -6.3765 | **0.0095817** | -2.75407 | 0.53975 |
| *LINC00486* | -0.16172 | -4.9535 | **0.0077556** | -0.64792 | 0.84838 |
| *PLIN4* | -0.12386 | -4.2955 | **0.041997** | 1.98906 | 0.66506 |
| *PPP1R16B* | -0.20240 | -16.2943 | **0.00082273** | -11.60958 | **0.051661** |
| *TLK1* | -0.12949 | -5.83659 | **0.033440** | 1.43766 | 0.74391 |
| *UBE2F* | -0.12657 | -6.0971 | **0.037673** | -2.13847 | 0.66633 |

^1^ Correlation Coefficient

^2^ OLS coefficient (Marginal)

^3^ OLS pvalue (Marginal)

^4^ OLS coefficient (Joint)

^5^ OLS pvalue (Joint)

# **Supplementary Table 8.** Regression coefficients and p-values from PROGRESS validation dataset using Body Mass Index.

We used the genes selected in SIBSIGHT as predictors of BMI and performed a linear regression to evaluate their association with PROGRESS BMI scores.

| Name | coefcor^1^ | coeflm^2^ | pvalue^3^ | jointcoeff^4^ | jointpvalue^5^ |
| --- | --- | --- | --- | --- | --- |
| *HRH2* | -0.013228 | -0.54713 | 0.82871 | -0.87206 | 0.80371 |
| *PLIN4* | -0.045939 | -1.0474 | 0.45220 | -5.2560 | **0.021123** |
| *PPP1R16B* | -0.036011 | -1.9059 | 0.55575 | -2.4344 | 0.460997 |
| *UBE2F* | 0.079208 | 2.5085 | 0.19445 | 8.7311 | **0.00401597** |

^1^ Correlation Coefficient

^2^ OLS coefficient (Marginal)

^3^ OLS pvalue (Marginal)

^4^ OLS coefficient (Joint)

^5^ OLS pvalue (Joint)

#

# **Supplementary Table 9.** Regression coefficients and p-values from PROGRESS validation dataset using Weight-for-Length.

We used the genes selected in SIBSIGHT as predictors of weight-for-length ratio and performed a linear regression to evaluate their association with PROGRESS weight-for-length ratio scores.

| Name | coefcor^1^ | coeflm^2^ | pvalue^3^ | jointcoeff^4^ | jointpvalue^5^ |
| --- | --- | --- | --- | --- | --- |
| *ABHD16A* | -0.06395 | -4.1153 | 0.29511 | 4.7511 | 0.57617 |
| *CCDC28A-AS1* | -0.039994 | -1.8159 | 0.51286 | 9.1543 | 0.20502 |
| *CCNG2* | -0.05653 | -1.02607 | 0.35480 | 0.73173 | 0.70032 |
| *DNASE1* | -0.085808 | -4.1959 | 0.15972 | -2.4685 | 0.60966 |
| *ERP27* | -0.064821 | -0.97326 | 0.28856 | -0.59163 | 0.73663 |
| *FAM168B* | 0.023452 | 0.47367 | 0.70125 | 0.640296 | 0.64617 |
| *KCTD20* | 0.020412 | 1.4286 | 0.73847 | 4.1064 | 0.49032 |
| *LINC00486* | -0.063162 | -1.7857 | 0.30110 | -1.5726 | 0.64533 |
| *LMTK2* | 0.0037663 | 0.22581 | 0.95088 | 2.6310 | 0.59831 |
| *LOC100129931* | 0.044005 | 4.7732 | 0.47149 | 14.808 | 0.11446 |
| *NCAPH* | -0.05556 | -1.15995 | 0.36312 | -1.6378 | 0.23809 |
| *OBI1-AS1* | -0.075565 | -7.8813 | 0.21584 | -5.9929 | 0.61726 |
| *PLIN4* | -0.044307 | -1.4183 | 0.46844 | -6.7985 | 0.17025 |
| *PPP1R16B* | -0.076829 | -5.7089 | 0.20823 | -0.85031 | 0.93693 |
| *RASSF1* | -0.086701 | -5.0332 | 0.15540 | -5.8135 | 0.379667 |
| *RBM28* | -0.062905 | -3.29198 | 0.30308 | -3.2668 | 0.63397 |
| *RSF1* | -0.0046131 | -0.21401 | 0.939861 | -3.9159 | 0.44889 |
| *SLC6A2* | -0.067418 | -3.1597 | 0.26963 | -7.9535 | 0.20460 |
| *SMIM20* | -0.063760 | -4.1448 | 0.29654 | -13.016 | **0.042614** |
| *UBE2F* | 0.039684 | 1.7645 | 0.51614 | 9.3191 | **0.066160** |

^1^ Correlation Coefficient

^2^ OLS coefficient (Marginal)

^3^ OLS pvalue (Marginal)

^4^ OLS coefficient (Joint)

^5^ OLS p-value (Joint)

# **Supplementary Table 10**. Methylation Risk Score associations with phenotypes at later time points in the SIBSIGHT cohort.

Methylation risk scores are calculated with the phenotype at 6 months. These are the linear regression coefficients of the relationship of those scores with phenotypes later in life.

|  | Methylation Risk Score | Phenotype | Adjusted R^2^ | p-value |
| --- | --- | --- | --- | --- |
| Cord Blood | BMI | BMI @ 1year | 0.4189 | 1.726 x 10^-7^ |
|  | BMI | BMI @ 2years | 0.2503 | 2.03 x 10^-4^ |
|  | weight-for-length | Weight-for-length @ 1year | 0.5825 | 1.726 x 10^-10^ |
|  | weight-for-length | Weight-for-length @ 2years | 0.3716 | 3.247 x 10^-6^ |
|  | CWG | BMI @ 1year | 0.5431 | 1.415 x 10^-9^ |
|  | CWG | BMI @ 2years | 0.249 | 2.2115 x 10^-4^ |
|  | CWG | Weight-for-length @ 1year | 0.4125 | 5.185 x 10^-7^ |
|  | CWG | Weight-for-length @ 2years | 0.2401 | 2.8 x 10^-4^ |
| Placenta | CWG | BMI @ 1year | 0.4571 | 1.107 x 10^-7^ |
|  | CWG | BMI @ 2years | 0.1891 | 1.483 x 10^-3^ |
|  | CWG | Weight-for-length @ 1year | 0.3359 | 1.177 x 10^-5^ |
|  | CWG | Weight-for-length @ 2years | 0.183 | 1.774 x 10^-3^ |

##

# Supplementary Table 11. Methylation Risk Score Associations with phenotypes in the PROGRESS cohort.

Methylation risk scores were derived from the SIBSIGHT cohort. These scores were then calculated using methylation data from the PROGRESS cohort. The data shown here are the results of linear regressions of the MRS and phenotypes.

|  | Methylation Risk Score | Phenotype | Adjusted R^2^ | p-value |
| --- | --- | --- | --- | --- |
| Cord Blood | CWG | CWG | -0.002118 | 0.5108 |
|  |  | BMI @ 1 year | -0.001999 | 0.4744 |
|  |  | BMI @ 1.5 years | -0.004094 | 0.9081 |
|  |  | BMI @ 2 years | -0.002947 | 0.599 |
|  |  | Weight/Length @ 1 year | -0.004087 | 0.9338 |
|  |  | Weight/Length @ 1.5 years | -0.004038 | 0.8701 |
|  |  | Weight/Length @ 2 years | -0.001793 | 0.4551 |
|  | BMI | BMI @ 6 months | -0.001946 | 0.4919 |
|  |  | BMI @ 1 year | -0.00314 | 0.6274 |
|  |  | BMI @ 1.5 years | -0.003981 | 0.8406 |
|  |  | BMI @ 2 years | -0.00374 | 0.7731 |
|  | Weight/Length | Weight/Length @ 6 months | -0.001928 | 0.4897 |
|  |  | Weight/Length @ 1 year | -0.004101 | 0.9526 |
|  |  | Weight/Length @ 1.5 years | -0.004077 | 0.895 |
|  |  | Weight/Length @ 2 years | -0.002835 | 0.5815 |

# Supplementary Note 1. Stratified analysis based on sex

We separated the samples by sex and performed the same regression analysis on each group that we performed on the whole population. The small sample size caused problems when running the LASSO analyses, the analysis of weight-for length ratio gave no results in females, and the LASSO analyses overall were rarely converging and frequently not providing the characteristic “check mark”. The stratified analysis for placenta was also inconclusive in males.

We found no overlap in the associated genes between the two sexes.

In cord blood :

- In males one gene was found significantly associated with all three outcomes: *CBLIF*. Two genes were significant in males and in the nonstratified analyses : *LOC105369771* was associated with CWG in males and with weight-for-length in the whole sample; and *ABRAXAS1* was associated with weight-for length in males and in the whole sample.
- In females, we *UBE2F* was associated with both CWG and BMI. We also found that *LINC00486* was associated with CWG and weight-for-length in the whole sample and CWG in females.

In placenta, one gene was found to be significant in common between the stratified analysis and the whole sample : *OR4D1* was associated with CWG in the whole sample and with weight-for-length in males. Additionally, in males, three genes were found to be associated with both BMI and weight-for-length ratio: *HRAT5*, *PRRX1*, and *MAPKAPK2*. In the literature, *HRAT5* has been found to contain variants associated with waist circumference[^1,2^](https://paperpile.com/c/4x77ig/qTfZ+vjFA), and *CBLIF*, *OR4D1*, *PRRX1*, and *MAPKAPK2* contain common variants associated with body height[^3^.](https://paperpile.com/c/4x77ig/1NLU)

Overall, the small sample size of this stratified analysis, and the high p-values, lead to a low confidence in these results. It would be interesting to repeat them in larger samples in the future.

## Females - Cord Blood

### Genes associated with CWG

| Name | function | Marginal CorrelationCoefficient | p-value |
| --- | --- | --- | --- |
| *HES5* | hes family bHLH transcription factor 5 | 12.34 | 2E-02 |
| *RHNO1* | RAD9-HUS1-RAD1 interacting nuclear orphan 1 | 46.90 | 4E-03 |
| *RSRP1* | arginine and serine rich protein 1 | 33.93 | 4E-02 |
| *LINC00486* | long intergenic non-protein coding RNA 486 | -90.42 | 1E-03 |
| *LINC02687* | long intergenic non-protein coding RNA 2687 | 28.26 | 2E-02 |
| *RFX7* | regulatory factor X7 | 15.54 | 3E-03 |
| *LOC107984471* | unknown | 11.23 | 1E-02 |
| *CALN1* | calneuron 1 | -127.70 | 5E-03 |
| *OBI1* | ORC ubiquitin ligase 1 | 20.68 | 2E-03 |
| *CD8B* | CD8b molecule | -53.75 | 1E-02 |
| *LOC105371024* | unknown | 77.40 | 7E-05 |
| *FBXW8* | F-box and WD repeat domain containing 8 | -79.79 | 2E-03 |
| *NKAIN2* | sodium/potassium transporting ATPase interacting 2 | 30.21 | 2E-01 |
| *LOC105376306* | uncharacterized LOC105376306 | -33.38 | 4E-02 |
| *UBE2F* | ubiquitin conjugating enzyme E2 F putative | 93.30 | 3E-05 |

### Genes associated with BMI

| Name | function | Marginal Correlation Coefficient | p-value |
| --- | --- | --- | --- |
| *LOC107984471* | unknown | 11.23 | 1E-02 |
| *LOC105371024* | unknown | 77.40 | 7E-05 |
| *LOC107986526* | unknown | 8.24 | 2E-02 |
| *LINC02275* | long intergenic non-protein coding RNA 2275 | 33.09 | 1E-01 |
| *UBE2F* | ubiquitin conjugating enzyme E2 F putative | 93.30 | 3E-05 |

### Genes associated with the weight-for-length ratio

Inconclusive results

## Males - Cord Blood

### Genes associated with CWG

| Name | function | Marginal Correlation Coefficient | p-value |
| --- | --- | --- | --- |
| *TDRP* | testis development related protein | 6.73 | 3E-01 |
| *G6PC* | glucose-6-phosphatase catalytic subunit | -29.42 | 1E-01 |
| *LOC105369771* | unknown | 34.24 | 2E-03 |
| *LOC105374598* | unknown | -19.32 | 3E-03 |
| *CBLIF* | cobalamin binding intrinsic factor | -8.39 | 5E-03 |
| *PIAS1* | protein inhibitor of activated STAT 1 | 117.01 | 4E-02 |
| *UFL1-AS1* | unknown | 4.70 | 8E-01 |
| *CLASP1* | cytoplasmic linker associated protein 1 | -18.01 | 2E-01 |
| *CAPN9* | calpain 9 | 81.29 | 4E-03 |

### Genes associated with BMI

###

| Name | function | Marginal Correlation Coefficient | p-value |
| --- | --- | --- | --- |
| *CBLIF* | cobalamin binding intrinsic factor | -8.39 | 5E-03 |

### Genes associated with the weight-for-length ratio

| Name | function | Marginal Correlation Coefficient | pvalue |
| --- | --- | --- | --- |
| *RILP* | Rab interacting lysosomal protein | 21.56 | 4E-02 |
| *LINC02249* | long intergenic non-protein coding RNA 2249 | -15.13 | 6E-02 |
| *CBLIF* | cobalamin binding intrinsic factor | -8.39 | 5E-03 |
| *STAP1* | signal transducing adaptor family member 1 | 27.14 | 1E-03 |
| *CEP295NL* | CEP295 N-terminal like | 44.28 | 4E-02 |
| *LOC105378816* | unknown | -4.65 | 1E-01 |
| *ABRAXAS1* | abraxas 1%2C BRCA1 A complex subunit | -64.20 | 4E-03 |
| *NCAPH* | non-SMC condensin I complex subunit H | -62.61 | 1E-02 |
| *UFL1-AS1* | unknown | 4.70 | 8E-01 |
| *GPR15* | G protein-coupled receptor 15 | -9.47 | 1E-01 |
| *RBM18* | RNA binding motif protein 18 | -4.71 | 3E-02 |
| *OSGEPL1* | O-sialoglycoprotein endopeptidase like 1 | 5.68 | 1E-01 |
| *PPIL3* | peptidylprolyl isomerase like 3 | -0.42 | 9E-01 |

## Females - Placenta

### Genes associated with CWG

| Name | function | Marginal Correlation Coefficient | p-value |
| --- | --- | --- | --- |
| *ZNF491* | zinc finger protein 491 | -34.72 | 6E-02 |
| *ARHGDIB* | Rho GDP dissociation inhibitor beta | -7.57 | 7E-01 |
| *GDF15* | growth differentiation factor 15 | 0.62 | 9E-01 |
| *LOC105376644* | unknown | 3.16 | 5E-01 |
| *DCAKD* | dephospho-CoA kinase domain containing | 30.74 | 2E-01 |
| *BRD1* | bromodomain containing 1 | 16.02 | 2E-01 |
| *LOC105377296* | unknown | 5.59 | 4E-01 |
| *LOC112268196* | unknown | -14.85 | 1E-01 |
| *LOC107986838* | unknown | -5.41 | 5E-01 |
| *LOC105377458* | uncharacterized LOC105377458 | -6.86 | 3E-01 |
| *PSMD4* | proteasome 26S subunit%2C non-ATPase 4 | -28.15 | 3E-01 |
| *LOC105377544* | unknown | -5.76 | 6E-01 |

### Genes associated with BMI

Inconclusive results

### Genes associated with the weight-for-length ratio

Inconclusive results

## Males - Placenta

### Genes associated with CWG

Inconclusive results

### Genes associated with BMI

| Name | function | Marginal Correlation Coefficient | p-value |
| --- | --- | --- | --- |
| *HRAT5* | heart tissue-associated transcript 5 | 2.25 | 8E-01 |
| *LINC02523* | long intergenic non-protein coding RNA 2523 | 24.57 | 1E-01 |
| *PRRX1* | paired related homeobox 1 | 5.81 | 3E-01 |
| *MAPKAPK2* | MAPK activated protein kinase 2 | -1.95 | 8E-01 |

### Genes associated with the weight-for-length ratio

| Name | function | Marginal Correlation Coefficient | p-value |
| --- | --- | --- | --- |
| *HRAT5* | heart tissue-associated transcript 5 | 2.25 | 8E-01 |
| *OR4D1* | olfactory receptor family 4 subfamily D member 1 | -4.43 | 6E-01 |
| *PRRX1* | paired related homeobox 1 | 5.81 | 3E-01 |
| *RAB24* | RAB24%2C member RAS oncogene family | -17.99 | 4E-01 |
| *MAPKAPK2* | MAPK activated protein kinase 2 | -1.95 | 8E-01 |

#

# Supplementary Note 2. Location of associated CpG sites within the associated genes

Once we identified genes associated with different weight outcomes, we repeated the Lasso analyses using the individual CpG sites located within these genes. We identified a subset of CpG sites driving the association in certain genes associated with the CWG and BMI score in the cord blood, and with the CWG score in the placenta. Other genes did not appear to have significantly associated CpGs, indicating that the overall methylation of the gene drives the association to the weight outcome. We mapped the location of the CpGs on the genes but did not identify patterns when looking at individual outcomes. We did notice that the same CpGs drive gene associations with CGW and BMI scores (Sup. Note 2 Figure 1).


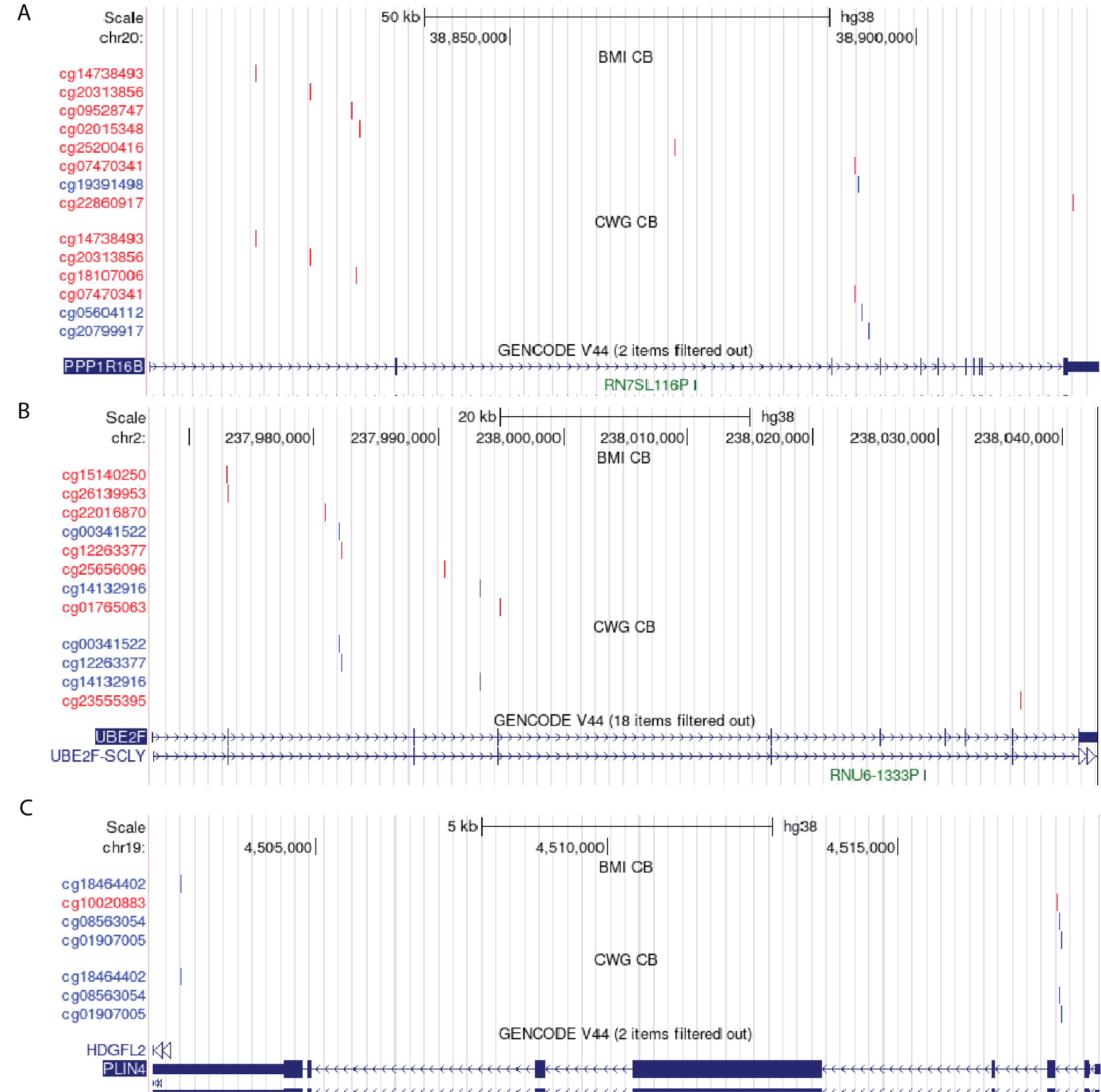


**Supplementary Note 2 Figure 1.** Mapping of CpG sites associated with BMI and CWG in cord blood. In blue: sites with marginal correlation p-value below 10^-2^ . In red: sites with marginal correlation p-value above 10^-2^. The genes shown here are the genes that were associated with BMI and CWG and contain CpG sites associated with BMI and CWG. (**A**) CpG sites mapping to *PPP1R16B*. (**B**) CpG sites mapping to *UBE2F*. (**C**) CpG sites mapping to *PLIN4*.

###

### CpGs Associated with CWG in Cord Blood

| CpG | Chromosome | Position | Gene | Marginal Correlation Coefficient | p-value | Region |
| --- | --- | --- | --- | --- | --- | --- |
| cg01907005 | chr19 | 4517804 | *PLIN4* | 18.94 | 4E-04 | intron |
| cg08563054 | chr19 | 4517763 | *PLIN4* | 10.57 | 7E-03 | intron |
| cg18464402 | chr19 | 4502678 | *PLIN4* | 12.23 | 6E-03 | 3'UTR |
| cg03035115 | chr16 | 21234122 | *ANKS4B* | -6.24 | 7E-03 | intron |
| cg06207722 | chr16 | 21244064 | *ANKS4B* | -3.11 | 3E-01 | intron |
| cg26937434 | chr16 | 21245151 | *ANKS4B* | -11.11 | 2E-03 | intron |
| cg05604112 | chr20 | 38893237 | *PPP1R16B* | -100.50 | 3E-03 | intron? |
| cg07470341 | chr20 | 38892365 | *PPP1R16B* | -6.03 | 3E-01 | intron? |
| cg14738493 | chr20 | 38818738 | *PPP1R16B* | -4.73 | 3E-01 | 5' UTR |
| cg18107006 | chr20 | 38831166 | *PPP1R16B* | -3.48 | 6E-02 | 5' UTR |
| cg20313856 | chr20 | 38825345 | *PPP1R16B* | -4.48 | 2E-01 | 5' UTR |
| cg20799917 | chr20 | 38894198 | *PPP1R16B* | -7.43 | 8E-03 | intron? |
| cg05167232 | chr6 | 138725317 | *CCDC28A-AS1* | 15.75 | 7E-03 | exon |
| cg08859635 | chr6 | 138745533 | *CCDC28A-AS1* | 25.74 | 3E-02 | intron |
| cg07070178 | chr3 | 183145849 | *LAMP3* | -20.76 | 6E-01 | intron |
| cg15439110 | chr3 | 183153901 | *LAMP3* | 12.87 | 9E-03 | intron |
| cg20696912 | chr3 | 183146011 | *LAMP3* | 21.78 | 4E-01 | intron |
| cg21200353 | chr3 | 183145532 | *LAMP3* | 29.76 | 2E-02 | intron |
| cg25737410 | chr3 | 183145478 | *LAMP3* | 18.38 | 7E-03 | intron |
| cg00341522 | chr2 | 237981972 | *UBE2F* | 9.98 | 1E-03 | intron |
| cg12263377 | chr2 | 237982246 | *UBE2F* | 6.15 | 8E-03 | intron |
| cg14132916 | chr2 | 237993322 | *UBE2F* | 17.31 | 1E-03 | intron |
| cg23555395 | chr2 | 238036564 | *UBE2F* | 5.12 | 9E-02 | intron |

### CpGs Associated with BMI in Cord Blood

| CpG | Chromosome | Position | Gene | Marginal Correlation Coefficient | pvalue | Region |
| --- | --- | --- | --- | --- | --- | --- |
| cg01907005 | chr19 | 4517804 | *PLIN4* | 26.01 | 8E-04 | intron |
| cg08563054 | chr19 | 4517763 | *PLIN4* | 16.92 | 3E-03 | intron |
| cg10020883 | chr19 | 4517722 | *PLIN4* | 13.45 | 1E-02 | intron |
| cg18464402 | chr19 | 4502678 | *PLIN4* | 16.99 | 9E-03 | 3'UTR |
| cg02015348 | chr20 | 38831576 | *PPP1R16B* | -2.70 | 6E-01 | 5' UTR |
| cg07470341 | chr20 | 38892365 | *PPP1R16B* | -8.64 | 3E-01 | intron |
| cg09528747 | chr20 | 38830619 | *PPP1R16B* | -75.93 | 6E-02 | 5' UTR |
| cg14738493 | chr20 | 38818738 | *PPP1R16B* | -9.10 | 2E-01 | 5' UTR |
| cg19391498 | chr20 | 38892846 | *PPP1R16B* | -9.96 | 1E-03 | intron |
| cg20313856 | chr20 | 38825345 | *PPP1R16B* | -8.47 | 7E-02 | 5' UTR |
| cg22860917 | chr20 | 38919351 | *PPP1R16B* | 4.67 | 6E-01 | 3'UTR |
| cg25200416 | chr20 | 38870290 | *PPP1R16B* | -7.79 | 7E-01 | intron |
| cg16256592 | chr5 | 175664221 | *HRH2* | 9.71 | 1E-01 | 5' UTR |
| cg17398003 | chr5 | 175665565 | *HRH2* | 44.09 | 8E-03 | 5' UTR |
| cg23004862 | chr5 | 175669020 | *HRH2* | 9.97 | 3E-02 | 5' UTR |
| cg00341522 | chr2 | 237981972 | *UBE2F* | 13.40 | 3E-03 | intron |
| cg01765063 | chr2 | 237994925 | *UBE2F* | 32.57 | 2E-01 | intron |
| cg12263377 | chr2 | 237982246 | *UBE2F* | 8.49 | 1E-02 | intron |
| cg14132916 | chr2 | 237993322 | *UBE2F* | 31.10 | 4E-05 | intron |
| cg15140250 | chr2 | 237973058 | *UBE2F* | -12.12 | 4E-01 | intron |
| cg22016870 | chr2 | 237980832 | *UBE2F* | 14.93 | 2E-01 | intron |
| cg25656096 | chr2 | 237990400 | *UBE2F* | 12.77 | 4E-01 | intron |
| cg26139953 | chr2 | 237973085 | *UBE2F* | -5.73 | 6E-01 | intron |

### CpGs Associated with CWG in Placenta

| CpG | Chromosome | Position | Gene | Marginal Correlation Coefficient | p-value | Region |
| --- | --- | --- | --- | --- | --- | --- |
| ch.1.1029351R | chr1 | 31734918 | *ADGRB2* | 12.95 | 7E-05 | intron |
| cg26534468 | chr17 | 35537229 | *LOC107985032* | -7.28 | 1E-04 | NA |
| cg01538301 | chr14 | 52837337 | *LOC105370500* | -2.38 | 2E-01 | 5' UTR |
| cg09208942 | chr14 | 52794647 | *LOC105370500* | -4.37 | 2E-03 | 5' UTR |
| cg20854374 | chr11 | 57747875 | *BTBD18* | -9.11 | 5E-05 | intron |
| cg01248878 | chr17 | 58155376 | *OR4D1* | 6.51 | 8E-04 | 5' UTR |
| cg13717532 | chr14 | 68972555 | *ACTN1* | -4.86 | 6E-02 | intron |
| cg18459449 | chr14 | 68976569 | *ACTN1* | -3.57 | 3E-01 | intron |
| cg23526087 | chr14 | 68973466 | *ACTN1* | -7.10 | 5E-03 | intron |
| cg26315918 | chr3 | 111793950 | *LOC107984087* | 9.29 | 3E-04 | intron |
| cg02620361 | chr7 | 115117424 | *LINC01393* | 8.44 | 3E-03 | intron |
| cg08532608 | chr7 | 141973378 | *TAS2R38* | -6.55 | 5E-05 | exon |
| cg01911613 | chr3 | 196325238 | *TM4SF19-TCTEX1D2* | -4.03 | 5E-02 | intron |
| cg12288267 | chr3 | 196318719 | *TM4SF19-TCTEX1D2* | -2.07 | 2E-02 | 3' UTR |
| cg22120738 | chr3 | 196326093 | *TM4SF19-TCTEX1D2* | -3.71 | 2E-02 | intron |

#

# Supplementary Note 3. Evaluating the methylation effects of genes previously identified to contain genetic variants important for childhood obesity

We evaluated the impact of genes previously identified as containing genetic variants associated with BMI[^4^](https://paperpile.com/c/4x77ig/FoQR). The first analysis we performed was a LASSO analysis to evaluate whether a subset of these genes was associated with the weight outcomes we studied. None of these LASSO analyses converged.

We then calculated the marginal correlation of each individual gene with each weight outcome: CWG, BMI, and weight-for-length (WL), in both cord blood (CB) and placenta (P). We did not have methylation data for *FOXP1*, *LINC00557*, and *PPP1R3A*. Overall, the p-values were high and suggest that there is no correlation between the methylation of these genes and weight outcomes.

| **Gene names** | **Coeff**  CWG  CB | **P-value**  CWG  CB | **Coeff**  BMI  CB | **P-value**  BMI  CB | **Coeff**  WL  CB | **P-value**  WL  CB | **Coeff**  CWG  P | **P-value**  CWG  P |
| --- | --- | --- | --- | --- | --- | --- | --- | --- |
| *DNAJC6* | -0.91 | 9E-01 | 0.19 | 1E+00 | 7.87 | 6E-01 | -6.87 | 4E-01 |
| *ZNF648* | 0.46 | 1E+00 | -7.62 | 6E-01 | -0.57 | 1E+00 | 0.64 | 8E-01 |
| *NR5A2* | 22.86 | 2E-01 | 31.65 | 2E-01 | 15.97 | 4E-01 | -9.70 | 2E-01 |
| *NAV1* | 8.10 | 7E-01 | 11.90 | 7E-01 | 13.01 | 6E-01 | -9.03 | 3E-01 |
| *RHOU* | -28.26 | 3E-01 | -13.88 | 7E-01 | -6.19 | 8E-01 | -4.91 | 8E-01 |
| *NOL10* | -61.31 | 9E-02 | -67.94 | 2E-01 | -41.43 | 3E-01 | -2.23 | 8E-01 |
| *FOXP1* | -40.20 | 2E-01 | -27.39 | 6E-01 | -2.24 | 9E-01 | -1.45 | 9E-01 |
| *TNIP3* | -1.29 | 7E-01 | 1.02 | 9E-01 | 1.06 | 8E-01 | -0.08 | 1E+00 |
| *FABP6* | 2.68 | 8E-01 | 1.95 | 9E-01 | -8.30 | 4E-01 | -2.70 | 3E-01 |
| *MTCH1* | -14.60 | 3E-01 | -10.79 | 6E-01 | -0.45 | 1E+00 | 9.27 | 9E-02 |
| *RNF144B* | -35.58 | 9E-02 | -45.43 | 1E-01 | -28.77 | 2E-01 | -2.12 | 8E-01 |
| *PTPRN2* | 73.31 | 2E-01 | 129.23 | 1E-01 | 143.02 | 3E-02 | 10.55 | 1E-01 |
| *POR* | -56.98 | 8E-02 | -71.28 | 1E-01 | -63.12 | 7E-02 | -10.03 | 2E-01 |
| *OLFM1* | -6.16 | 8E-01 | -8.53 | 8E-01 | 7.59 | 7E-01 | -0.75 | 9E-01 |
| *BRINP1* | -10.77 | 1E-01 | -15.57 | 1E-01 | -12.71 | 1E-01 | -0.13 | 1E+00 |
| *RBFOX1* | -51.97 | 2E-01 | -126.65 | 3E-02 | -78.68 | 7E-02 | 17.08 | 5E-02 |
| *DNAH9* | 5.58 | 8E-01 | 24.60 | 5E-01 | 25.20 | 4E-01 | -9.38 | 5E-01 |
| *BAIAP2* | 2.43 | 9E-01 | 4.12 | 9E-01 | 0.76 | 1E+00 | -9.84 | 2E-01 |
| *TGIF1* | -38.99 | 3E-01 | -69.72 | 2E-01 | -65.30 | 1E-01 | 12.17 | 5E-01 |
| *TIAM1* | -20.09 | 4E-01 | -44.69 | 2E-01 | -27.10 | 2E-01 | 7.76 | 2E-01 |
| *ELFN2* | 10.34 | 8E-01 | 27.25 | 6E-01 | -7.67 | 9E-01 | -10.86 | 4E-01 |

1. [Zhu, Z. *et al.* Shared genetic and experimental links between obesity-related traits and asthma subtypes in UK Biobank. *J. Allergy Clin. Immunol.* **145**, 537–549 (2020).](http://paperpile.com/b/4x77ig/qTfZ)

2. [Christakoudi, S., Evangelou, E., Riboli, E. & Tsilidis, K. K. GWAS of allometric body-shape indices in UK Biobank identifies loci suggesting associations with morphogenesis, organogenesis, adrenal cell renewal and cancer. *Sci. Rep.* **11**, 10688 (2021).](http://paperpile.com/b/4x77ig/vjFA)

3. [Yengo, L. *et al.* A saturated map of common genetic variants associated with human height. *Nature* **610**, 704–712 (2022).](http://paperpile.com/b/4x77ig/1NLU)

4. [Craig, S. J. C. *et al.* Constructing a polygenic risk score for childhood obesity using functional data analysis. *Econ. Stat.* **25**, 66–86 (2023).](http://paperpile.com/b/4x77ig/FoQR)
